# Computational Models of Consumer Confidence from Large-Scale Online Attention Data: Crowd-Sourcing Econometrics

**DOI:** 10.1371/journal.pone.0120039

**Published:** 2015-03-31

**Authors:** Xianlei Dong, Johan Bollen

**Affiliations:** 1 School of Economics and Management, Beijing University of Technology, Beijing, China; 2 School of Informatics and Computing, Indiana University, Bloomington, United States of America; University of Warwick, UNITED KINGDOM

## Abstract

Economies are instances of complex socio-technical systems that are shaped by the interactions of large numbers of individuals. The individual behavior and decision-making of consumer agents is determined by complex psychological dynamics that include their own assessment of present and future economic conditions as well as those of others, potentially leading to feedback loops that affect the macroscopic state of the economic system. We propose that the large-scale interactions of a nation's citizens with its online resources can reveal the complex dynamics of their collective psychology, including their assessment of future system states. Here we introduce a behavioral index of Chinese Consumer Confidence (C3I) that computationally relates large-scale online search behavior recorded by Google Trends data to the macroscopic variable of consumer confidence. Our results indicate that such computational indices may reveal the components and complex dynamics of consumer psychology as a collective socio-economic phenomenon, potentially leading to improved and more refined economic forecasting.

## Introduction

The growth of most modern economies is driven by consumer spending [1]. Therefore, consumer confidence levels can have significant effects on economic growth. Consumer Confidence Indices (CCI) are designed to measure the degree of confidence that consumers have with respect to the state of the economic system. The basis for many CCIs lies in behavioral science where evidence has accumulated that individual consumer behavior is influenced by a number of emotional and social factors [2, 3] that interact with the consumer agents’ socio-economic context. In other words, the emotional state of consumers as well as their assessment of that of other consumers will shape their subsequent individual consumption patterns [4, 5]. In the aggregate, as consumers collectively lose or gain confidence in the state of the economy, this is assumed to affect their collective consumption patterns and thus economic growth yielding a complex interaction between consumer confidence and economic conditions. This interplay between the complex behavior of individual agents and the emergent properties of their collective behavior is analogous to those seen in many other large-scale socio-technical systems [6, 7].

Accurate, valid, and timely measures of consumer confidence are thus of pivotal importance to policy-makers and econometric forecasting. However, as a social and abstract construct “consumer confidence” is difficult to measure. Researchers have turned to social science methods such as surveys and questionnaires which are expensive and time-consuming to conduct, and are possibly subject to a number of personal, cultural, and social biases, e.g. social conformity bias [8] which will confound measures of consumer confidence with cultural and linguistic propensities to divulge or withhold accurate information concerning one’s level of confidence. The latter also makes it difficult to compare consumer confidence across different linguistic and cultural regions.

Here we investigate a computational approach that leverages large-scale search engine query volumes to gauge consumer confidence. We start from the assumption that search engine volumes reflect the issues that a population is contemporaneously pre-occupied with [9], congruent with recent work in the area of market modeling [10–14]. Hence, consumer confidence may be manifested in the volume of certain web searches such as “taxes”, “investment”, and “stocks”, but not others, e.g. “cloud” and “cat”. We focus on China since it provides an interesting case for Consumer Confidence studies given its unique linguistic and cultural background, and the important role that the consumption patterns of its burgeoning middle-class are now playing in the global economy [15].

We obtain Google query volume time series for a number of Chinese characters that are likely to express various facets of Chinese Consumer Confidence given their use in existing surveys of consumer confidence in China. Using a principal component analysis, we isolate the queries that are the main indicators of Chinese consumer confidence [16], and define a Chinese Consumer Confidence Index (C3I) from a linear combination of the respective search volume data. We cross-validate the C3I against existing gauges of consumer confidence, demonstrating its ability to offer an accurate, timely, and informative view on consumer confidence in a region that has been historically underserved with regards to econometric indices. Our results indicate that the C3I yields new information on the nature of Chinese Consumer Confidence. Our work may thus contribute to the science of modeling the social construct of consumer confidence and its socio-economic correlates that shape the emergent properties of economies as large-scale socio-technical systems [7].

## Materials and Methods

In our investigation we rely on the following data sources:

1. Consumer Confidence data from the Chinese Consumer Confidence Index (CCI) and the Economist’s Confidence Questionnaire (ECQ) surveys for the period under consideration.
2. Google Trend data for a specific number of search queries corresponding to the same time period.

Given the different construction of the CCI and ECQ, we use the first as an official indicator of Chinese Consumer Confidence and the latter as a source from which to extract consumer confidence topics that are subsequently translated into Google queries.

### Chinese Consumer Confidence Index (CCI) survey

**Fig 1:**
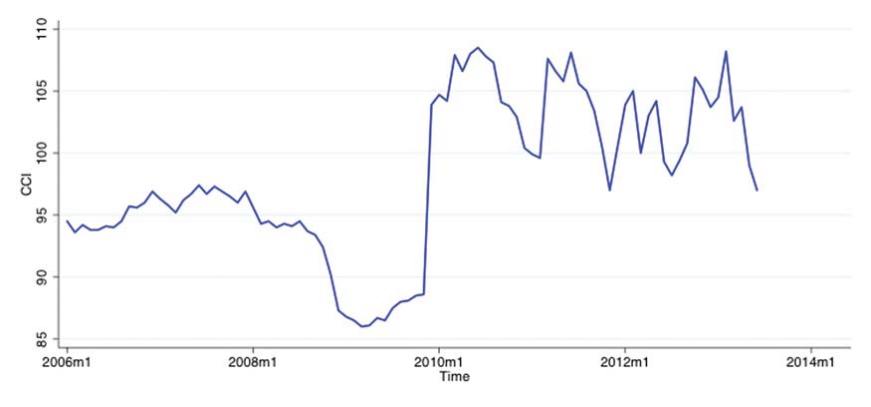
Monthly time series of Chinese CCI provided by the National Bureau of Statistics of China for the period of January 2006 to June 2013.

The Chinese Consumer Confidence Index (CCI) is reported by the National Bureau of Statistics of China (NBSC) on a monthly basis. Its methodology consists of asking 3,500 individuals (after November, 2009) about their confidence levels of the present and the future. It consists of a questionnaire of about 5 simple questions each pertaining to what is assumed to be a specific component of consumer confidence, e.g. “How do you see your current employment conditions?”. Subjects’ responses are recorded on a 5-point scale. We obtained historical monthly data of Chinese CCI from National Bureau of Statistics of China for the period of January 2006 to June 2013, i.e. 90 months, as shown in Fig 1. It must be noted that the CCI numbers reported by the NBSC may be affected by changing data normalization practices and other adjustments over time [17].

### Economist’s Confidence Questionnaire (ECQ) topic extraction

The CCI is designed to be succinct and fast to administer. Hence it consists of short questions designed to be answered in terms that are directly evaluative of the question, e.g. “How do you see your current employment conditions?” answered by either “positive” or “negative”. However, we are looking to model the notion of Chinese Consumer Confidence as exhaustively as possible so we can determine its correlates in online indicators.

The Economist’s Confidence Questionnaire (ECQ) contains 31 open questions such as “What do you presently consider the greatest threat to the Chinese economy?”, with a number of possible responses provided that can range from a few items to more than 15. Given the more open and exhaustive nature of the ECQ we manually extract the core topics of the ECQ’s questions and answers, and corresponding Chinese characters, to define an initial set of terms that can be reliably transformed to specific Google search queries. The volume of the latter are then taken to indicate the level of online attention with respect to that particular topic. For example, ECQ Question 13 is “How do you think the dollar value may change in the next 6 months?”. From this question we manually extract the Chinese character for “dollar trend”, and add it to the set of topics that we deem to be indicative of consumer confidence. We then retrieve Google Trend data for each such individual topic.

**Fig 2:**
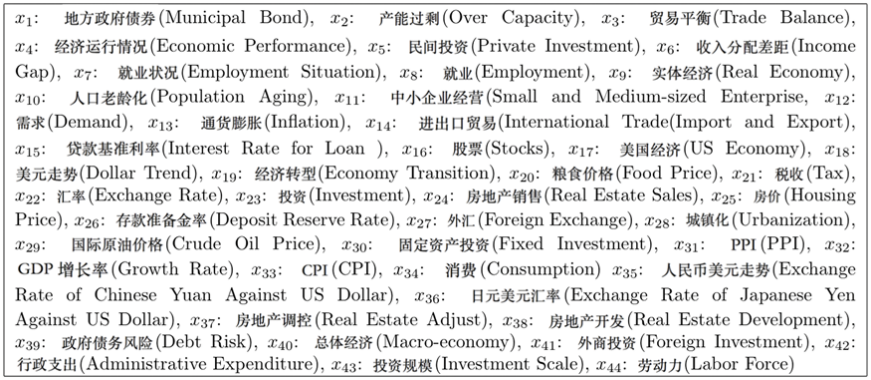
Topics and Variable Names extracted from CCI survey. Note: only the first 34 topics (*x*_1_-*x*_34_) are used as variables in our model since other topics did not have sufficient query volumes in Google Trends.

As shown in S1 Table (Supplementary materials) and Fig 2, we extract a total of 44 topics from the ECQ’s questions ranging from large-scale macro-economic concepts such as “inflation” to more personal notions such as “food price”. Out of those 44 topics, only 34 have sufficient Google query volumes and are thus included as variables in our later analysis.

### Google Trends data

**Fig 3:**
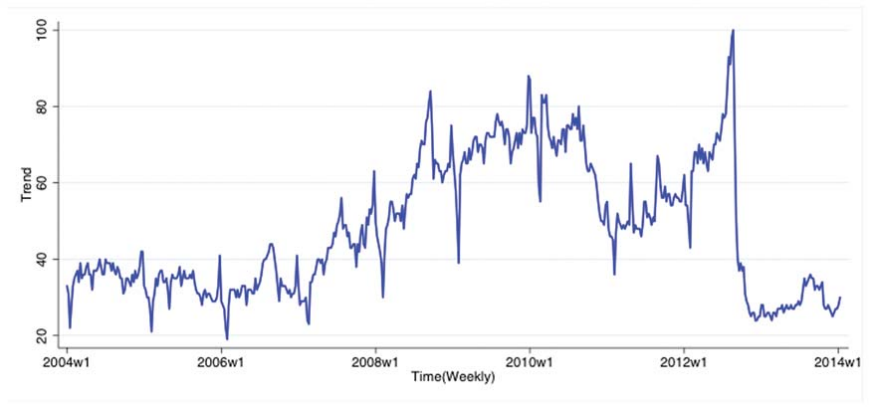
Google Trends graph showing weekly fluctuations of search volume for “Hao” (en: “good”).

Google Trends (www.google.com/trends/) is an online service offered by the Google search engine; it allows researchers to retrieve weekly/monthly normalized search volume data for any user-provided search query, provided the query has non-zero search volume. For example, a user can enter the query “good” and Google Trends will return a weekly time series whose values represent the volume of searches for that query recorded by Google in that period of time on a weekly basis. An example of the Google Trends data for the Chinese character “Hao” (en: “good”) is shown in Fig 3.

As such we obtain Google Trends data for the 34 above mentioned topics that produce non-zero search volumes from January 2006 to June 2013 thereby matching the date range of our CCI data. Since Google Trends data can be weekly and CCI data is released monthly, we convert all weekly Google Trends time series to monthly time series by means of a 4-week moving average. Since some months are longer than 4 weeks, where necessary, we move data points at the end of the month’s last week to the next month.

### Methodological overview

Our research objectives are four-fold:

1. We model Chinese Consumer confidence from the covariances between 34 ECQ topic time series
2. We define a new Chinese Consumer Confidence Index (C3I) based on the principal components of (1)
3. We compare our C3I to the CCI using a stepwise regression model that fits the C3I components to the CCI, including a determination of whether or not one indicator leads the other.
4. We conduct a preliminary test of our model against new Google Trends data that was not included in the original data used model construction (July 2013 to May 2014).

**Fig 4:**
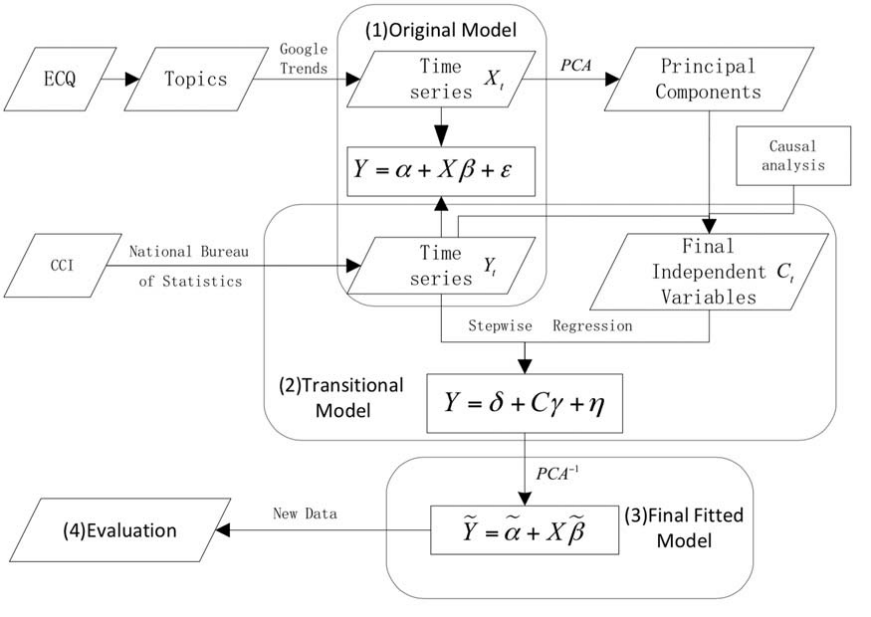
Methodological overview. (a) We study the relationship between China’s official CCI data (*Y*) and Google Trends Data (*X*). (b) We use a PCA to determine the principal components of *X*, followed by a Granger test and VAR to determine the lead or lag relations between *X* and *Y*. (c) *PCA* ^−1^ denotes the inverse operation of PCA to obtain the fitted values of our original model.

In Fig 4 we show an overview of our multi-phased methodology which is further explained in subsequent sections.

## Results and Discussion

### Principal Component Analysis of ECQ topic covariances

Each of the 34 Google trends time series (corresponding to the ECQ questionnaire topics) can be taken as independent variables, representing a certain facet of consumer confidence. However, we need to determine the degree of multicollinearity to investigate whether each variable independently represents consumer confidence, and to ensure the validity of later regression models used to fit a potential C3I based on these 34 independent variables to the CCI.

**Fig 5:**
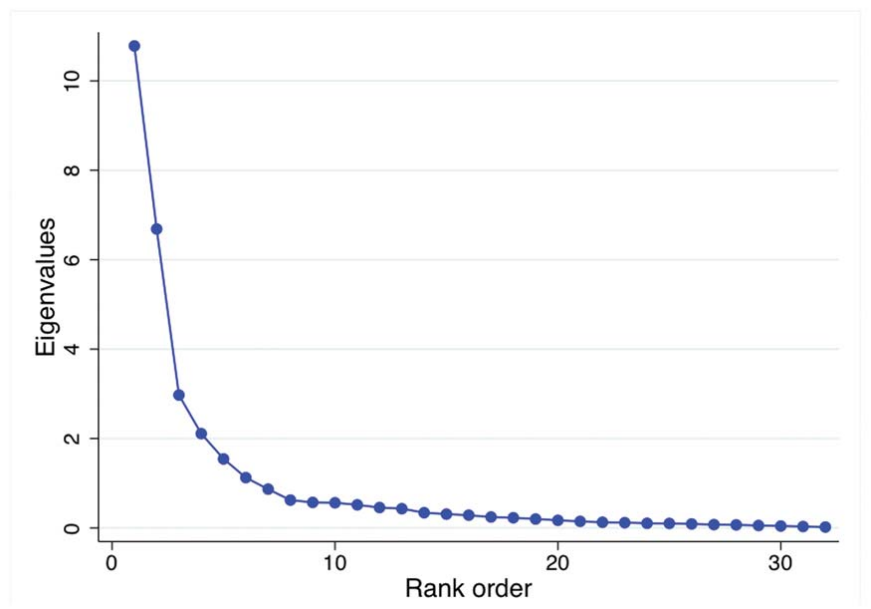
Scree plot showing the eigenvalue distribution of our Principal Component Analysis of 34 Google trends time series.

Therefore we perform a principal components analysis (PCA) [18] to ensure the orthogonality of our components and to avoid the issue of multicollinearity in future regression models. Furthermore, this procedure reduces dimensionality and may provide information on the underlying components of the covariances of our 34 Google trends time series. We list the 10 highest ranked components with their loadings in Table 1 and provide a scree plot in Fig 5. A Kaiser-Meyer-Olkin Measure of Sampling Adequacy (KMO) test [19] and Squared Multiple Correlation (SMC) test [20] indicate that the PCA was indeed a suitable procedure with the large majority of values well above 0.8 (1.0 is optimal).

**Table 1:** Principal Components of our 34 Google trends time series with proportion of variance covered.

Component	Proportion	Cumulative
1	0.310	0.310
2	0.219	0.529
3	0.088	0.616
4	0.071	0.687
5	0.047	0.734
6	0.033	0.767
7	0.030	0.797
8	0.027	0.824
9	0.021	0.845
10	0.018	0.863

Judging from the scree plot, we arbitrarily retain the first 9 PCA components since they represent the majority of information on the original topic covariances (about 85%), thus ensuring we retain all relevant information for accurate modeling. Not all 9 components need to be included in our transitional model since each carries increasingly less information. In fact, whether we choose 8, 9, or 10 components should be of little significance to our transitional model.

We project our topic variables on the selected 9 components, (*C*_1_, *C*_2_, …, *C*_9_), and define

$$C_{i,t}=X_{t}^{T}\times c_{i\in \{1,2,\ldots ,9\}}$$

where *X* = (*x*_1_, *x*_2_, …, *x*_34_)^*T*^ refers to our 34 topic time series and *c*_*i*_ refers to the entries of the 9 component vectors as listed in Table 2.

**Table 2:** Entries of the first 9 PCA components.

Components									
Variables	*c*_1_	*c*_2_	*c*_3_	*c*_4_	*c*_5_	*c*_6_	*c*_7_	*c*_8_	*c*_9_
*x*_1_	-0.166	0.018	0.196	0.047	-0.042	-0.286	-0.111	0.515	-0.192
*x*_2_	-0.030	-0.022	0.277	0.329	-0.085	0.476	0.263	-0.164	-0.052
*x*_3_	0.136	-0.031	0.264	-0.313	0.061	-0.309	0.328	-0.179	-0.060
*x*_4_	0.249	-0.069	-0.082	0.117	-0.056	0.046	0.061	0.075	0.011
*x*_5_	-0.052	0.124	0.335	0.135	-0.146	-0.177	0.038	0.245	0.418
*x*_6_	-0.133	-0.011	0.279	-0.057	0.440	-0.120	0.258	0.077	-0.215
*x*_7_	0.243	-0.067	0.029	0.064	-0.027	-0.114	0.058	0.043	-0.214
*x*_8_	0.254	-0.004	0.139	0.159	0.092	-0.001	-0.004	0.044	0.067
*x*_9_	-0.084	0.221	-0.062	0.240	0.112	-0.268	-0.092	-0.304	0.323
*x*_10_	0.094	-0.161	0.194	0.016	0.298	0.043	-0.357	0.054	0.491
*x*_11_	0.168	0.041	0.298	0.070	0.111	-0.232	0.230	-0.065	0.023
*x*_12_	0.294	-0.036	0.048	0.121	0.033	0.059	0.017	0.047	0.021
*x*_13_	0.142	0.170	0.141	-0.326	0.031	0.300	-0.064	0.054	-0.033
*x*_14_	0.288	-0.098	-0.048	0.036	-0.025	0.052	0.025	0.029	0.005
*x*_15_	0.049	0.283	-0.001	0.030	-0.067	-0.269	-0.210	-0.263	-0.139
*x*_16_	0.200	0.039	-0.001	-0.340	-0.004	-0.163	0.167	-0.294	0.119
*x*_17_	0.180	0.198	-0.107	0.167	0.275	-0.060	-0.147	0.055	-0.057
*x*_18_	-0.100	0.282	0.101	0.052	0.086	0.299	-0.010	-0.239	-0.004
*x*_19_	-0.073	0.241	0.260	0.214	-0.138	0.022	-0.050	0.020	-0.142
*x*_20_	0.063	0.309	0.033	-0.014	-0.047	-0.077	-0.076	0.045	-0.141
*x*_21_	0.277	-0.122	0.088	0.013	0.091	0.034	-0.101	0.016	-0.042
*x*_22_	0.008	0.267	0.034	0.010	0.319	0.109	-0.195	-0.124	-0.249
*x*_23_	0.236	0.142	-0.194	-0.014	0.022	0.041	0.167	0.194	-0.022
*x*_24_	0.250	-0.164	0.064	0.006	-0.021	0.038	-0.151	0.070	-0.130
*x*_25_	-0.048	0.214	0.338	0.042	-0.181	0.065	-0.013	-0.042	-0.051
*x*_26_	0.087	0.158	0.150	-0.343	-0.006	0.120	-0.328	0.121	0.121
*x*_27_	0.125	0.220	-0.271	0.132	0.204	-0.134	-0.063	-0.018	-0.067
*x*_28_	-0.154	-0.089	0.051	-0.050	0.555	0.149	0.093	0.107	0.067
*x*_29_	0.071	0.284	-0.118	0.112	-0.015	-0.007	0.152	0.365	0.046
*x*_30_	0.257	-0.052	0.096	0.105	0.016	0.058	-0.072	-0.026	-0.101
*x*_31_	-0.038	0.241	-0.241	-0.020	0.143	0.119	0.366	0.190	0.129
*x*_32_	0.128	0.236	-0.048	-0.071	-0.107	0.066	0.232	0.003	0.351
*x*_33_	0.088	0.214	0.045	-0.381	-0.099	0.131	-0.049	0.137	-0.027
*x*_34_	0.281	0.013	0.059	0.187	-0.030	0.015	0.051	-0.034	0.028

To avoid spurious regression results [21, 22], we must determine whether our time series are stationary (*I*(0)) or have co-integrated relationships. After we extract the 9 first components, we conduct a KPSS test [23] and an ADF test [24, 25] to check the variables’ stationarity. As shown in Table 3 only *C*_1_ is not stationary. We therefore define *DC*_1,*t*_ = *C*_1,*t*_−*C*_1,*t*−1_ (difference once), and find that it is stationary. Subsequently all the 10 variables (CCI, *DC*_1_, *C*_*i* ∈ {2, 3, ⋯, 9}_) are stationary.

**Table 3:** Stationary test results.

Variables	ADF (unit root) p value	KPSS (level stationary) Statistics (lag(11))	KPSS(trend stationary) Statistics (lag(11))
CCI	0.294	0.431	0.084
*C*_1_	0.781	0.834**	0.158*
*C*_2_	0.290	0.228	0.190*
*C*_3_	0.018*	0.226	0.101
*C*_4_	0.160	0.131	0.086
*C*_5_	0.003**	0.183	0.118
*C*_6_	0.005**	0.073	0.070
*C*_7_	0.000**	0.247	0.095
*C*_8_	0.019*	0.210	0.060
*C*_9_	0.000**	0.094	0.080

(1) Critical values of KPSS (level stationary)—10%: 0.347, 5%: 0.463, 2.5%: 0.574 1%: 0.739. (2) Critical values of KPSS (trend stationary)—10%: 0.119, 5%: 0.146, 2.5%: 0.176, 1%: 0.216. (3) * and ** mean rejecting *H*_0_ at the 5% level and 1% level respectively. (4) p-values equaling 0.000 indicate < 0.0005: we use the same notation throughout the paper.

### Model definition

After determining the principal components of our Google trend time series data, i.e. the components that best describe consumer confidence as indicated from Google query volume with respect to our 34 survey topics, we perform a Vector Auto-regression (VAR) [26] to determine the degree of auto-correlation in our CCI data. As shown in Table 4, we find a considerable degree of auto-correlation, indicating the necessity to include CCI at lag 1 and *C*_3_ at lag 2 as independent variables in future analysis. This finding is intuitive, since consumers may factor previous confidence into their assessment of future conditions along with other present information.

**Table 4:** Vector Auto-regression Results.

Dependent Var.: CCI		Coef.	Std. Err.	*z*	*P* > *z*
CCI	L1.	0.816	0.104	7.840	**0.000**
	L2.	0.025	0.112	0.220	0.823
*C*_2_	L1.	0.019	0.027	0.690	0.491
	L2.	-0.024	0.027	-0.870	0.387
*C*_3_	L1.	-0.031	0.024	-1.300	0.194
	L2.	0.065	0.026	2.500	**0.012**
*C*_4_	L1.	-0.004	0.027	-0.150	0.879
	L2.	0.006	0.026	0.240	0.807
*C*_5_	L1.	0.007	0.026	0.270	0.788
	L2.	-0.038	0.025	-1.500	0.134
*C*_6_	L1.	-0.021	0.024	-0.850	0.394
	L2.	0.043	0.024	1.800	0.072
*C*_7_	L1.	0.022	0.030	0.740	0.459
	L2.	-0.038	0.030	-1.270	0.204
*C*_8_	L1.	-0.001	0.023	-0.030	0.973
	L2.	-0.024	0.025	-0.960	0.338
*C*_9_	L1.	0.000	0.027	0.010	0.991
	L2.	0.003	0.025	0.110	0.909
*DC*_1_	L1.	-0.007	0.024	-0.290	0.774
	L2.	-0.003	0.019	-0.160	0.874

(1) L1 indicates a 1 month time series lag whereas L2 indicates a 2 month lag. (2) L.CCI and L2.*C*_3_ are significant with a low p-value in the VAR model, so they are added in the model as independent variables.

We conduct a Granger Causality test [27] between our independent variables, *C*_*i* ∈ {2, 3, ⋯, 9}_ and *DC*_1_ vs. one dependent variable, namely CCI_*t*_, to look for Granger-causative relationships between CCI and independent variables. The results in Table 5 indicate that independent variable *C*_2_ is Granger causative of the CCI. Results in behavioral science [28] indicate that people tend to discount older information in favor of newer information. We therefore choose variables that were lagged one and two units for testing. Refering to the results, we add *C*_2_ at lag 1 and lag 2 in our independent variables.

**Table 5:** Results of Granger Test of CCI vs. DC_1_ and *C*_{1, 2, ⋯, 9}_.

Equation	Excluded	*χ* ^2^	df	*p* > *χ* ^2^
CCI	*DC*_1_	0.093	2	0.595
CCI	*C*_2_	1.037	2	0.018*
CCI	*C*_3_	8.072	2	0.964
CCI	*C*_4_	0.073	2	0.111
CCI	*C*_5_	4.393	2	0.143
CCI	*C*_6_	3.892	2	0.431
CCI	*C*_7_	1.685	2	0.378
CCI	*C*_8_	1.945	2	0.991
CCI	*C*_9_	0.017	2	0.955
CCI	ALL	25.088	18	0.123

P-values marked * indicate the variable is Granger causative of the CCI at the 5% significance level.

A normalization of CCI data in reference to 1996 data [17] ended in November 2009 leading to an apparent discontinuity in the CCI data in 2009–2010 as shown in Fig 1. To determine whether our CCI data is biased by structural changes or not, we conduct as Structural Change test [29]. The results are summarized in Table 6; the null-hypothesis that no structural change occurred must be rejected. In other words, the results indicate a structural change is likely to have occurred in November 2009 possibly because of the use of new survey or normalization methods [17].

**Table 6:** Results of Structural Change Tests.

Structural Change Test: *Y* = *X* + *D*_0_ + *D*_*X*_	*H*_0_: no structural change
Chow Test [*K*, *N* − 2 * *K*] = 5.585	*p* > *F*(14, 60) 0.000**
Wald Test = 114.669	*p* > *χ* ^2^(43) 0.000**
Likelihood Ratio Test = 73.413	*p* > *χ* ^2^(43) 0.000**

Structural Change Tests indicate that we can reject the null-hypothesis of no structural change in CCI time series. P-values marked ** mean rejecting *H*_0_ at the 1% level.

As indicated in Table 6, all three tests imply there is a structural change in the time series, which may have resulted from the NBSC standardization in November 2009. We therefore add dummy variable *D* to all the independent variables of our model, where the first time period comprises 47 months and the second time period comprises 42 months.

$$\begin{array}{c} D_{t}=\left\{\begin{array}{c} 0,t\leq t_{0}\\ 1,t>t_{0} \end{array}\right. \end{array}$$ (1)

Then, our transitional model (i.e. Model 2 in Fig 4) can be written as follows:

$$\begin{array}{c} {\text{C3I}}_{t}=\left\{\begin{array}{c} \left(\alpha +D_{t}\gamma_{0}\right)+\left(\beta_{0}+D_{t}\delta \right){\text{CCI}}_{t-1}+\left(\beta_{1}+D_{t}\gamma_{1}\right)DC_{1}+\sum_{i=\{2-9\}}\left(\beta_{i}+D_{t}\gamma_{i}\right)C_{i,t}\\ +\left(\beta_{10}+D_{t}\gamma_{10}\right)C_{2,t-1}+\sum_{\begin{array}{c} i=\{11,12\}\\ j=\{2,3\} \end{array}}\left(\beta_{i}+D_{t}\gamma_{i}\right)C_{j,t-2}+\epsilon_{t} \end{array}\right. \end{array}$$ (2)

where *t*_0_ = 47.

### Results

We then proceed with a Stepwise Regression [30] as follows:

1. Set an appropriate significance level of 0.05.
2. Fit Eq 2 by Ordinary Least Squares (OLS).
3. If all the parameters pass the test, then stop, otherwise, proceed to step 4.
4. Select the variable with the lowest significance level, and drop it. Fit the new equation, minus the variable, by OLS.
5. Repeat step 3 and step 4, until all variables pass the test.

As shown in Table 7 the resulting model exhibits a good fit as indicated by a significant adjusted *R* ^2^ (0.923). We conduct a White Test [31] to determine whether the regression has heteroscedasticity. As indicated by the results shown in Table 8, this is the case. Therefore, we re-run our stepwise model with robust standard errors [32]. The results are shown in Table 9. The results improve considerably: all parameters pass the test and we observe an improved adjusted *R* ^2^. However, we must point out that although the use of robust standard errors improves the estimate, we can not guarantee that the regression has no heteroscedasticity.

**Table 7:** Regression results for model represented by Eq (2).

Parameters	Coef.	Std. Error	t-value	*P* > *t*
*α*	0.624	6.680	0.090	0.926
*γ*_0_	59.431	10.037	5.920	0.000
*β*_0_	0.992	0.072	13.840	0.000
*δ*	-0.540	0.103	-5.250	0.000
*γ*_2_	-0.112	0.026	-4.240	0.000
*γ*_3_	0.099	0.021	4.710	0.000
*γ*_5_	-0.074	0.017	-4.350	0.000
*γ*_6_	0.093	0.018	5.160	0.000
Adjusted *R* ^2^	=0.923			

Constant *α* (cf. Eq (2) at *t* ≤ 47) doesn’t pass the significance test.

**Table 8:** White test results.

*H*_0_: Homoskedasticity	
*χ* ^2^ = 44.330	*p* > *χ* ^2^ = 0.005

The results of a White Test indicate that we must reject the hypothesis of homoscedasticity in our model. Homoscedasticity is one of necessary conditions to consider the estimated parameters from classic OLS efficient estimators, i.e. Best Linear Unbiased Estimator property (BLUE) [33].

**Table 9:** Regression results for model represented by Eq (2).

Parameters	Coef.	Robust Std. Error	t-value	*P* > *t*
*α*	16.384	4.018	4.080	0.000
*β*_0_	0.832	0.041	20.100	0.000
*β*_4_	-0.016	0.006	-2.790	0.007
*β*_11_	-0.011	0.004	-2.730	0.008
*δ*	-0.380	0.102	-3.710	0.000
*γ*_0_	44.843	10.053	4.460	0.000
*γ*_2_	-0.117	0.026	-4.510	0.000
*γ*_3_	0.111	0.021	5.300	0.000
*γ*_5_	-0.086	0.019	-4.420	0.000
*γ*_6_	0.096	0.023	4.180	0.000
Adjust *R* ^2^	=0.933			

All parameters including *α* (Eq (2) with *t* ≤ 47) pass the test.

Using the regression results we can model C3I as shown in Eq (3).

$$\begin{array}{c} {\text{C3I}}_{t}=\left\{\begin{array}{c} 16.384+0.832{\text{CCI}}_{t-1}-0.016C_{4,t}-0.011C_{2,t-2},t\leq 47;\\ 61.227+0.452{\text{CCI}}_{t-1}-0.117C_{2,t}+0.111C_{3,t}\\ -0.016C_{4,t}-0.086C_{5,t}+0.096C_{6,t}-0.011C_{2,t-2},t>47. \end{array}\right. \end{array}$$ (3)

This fitted equation preserves the major components of the PCA (*C*_2_−*C*_6_) to avoid significant information loss. We can formulate our final fitted model using the original indices as shown in Eq (4).

$$\begin{array}{c} {\text{C3I}}_{t}=\left\{\begin{array}{c} t\leq 47:16.384+0.832{\text{CCI}}_{t-1}+X_{t}A+X_{t-2}C\\ t>47:61.227+0.452{\text{CCI}}_{t-1}+X_{t}B+X_{t-2}C \end{array}\right. \end{array}$$ (4)

where *X* ^*T*^ = (*x*_1_, *x*_2_, …, *x*_34_); and the entries of *A*, *B*, and *C* are provided in Table 10.

**Table 10:** Topic Effect on C3I according to matrix *A*, *B* and *C*.

Variables	A	B	C	Variables	A	B	C
*x*_1_	-0.001	-0.005	0.000	*x*_18_	-0.001	-0.001	-0.003
*x*_2_	-0.005	0.081	0.000	*x*_19_	-0.003	0.011	-0.003
*x*_3_	0.005	0.003	0.000	*x*_20_	0.000	-0.036	-0.003
*x*_4_	-0.002	0.006	0.001	*x*_21_	0.000	0.019	0.001
*x*_5_	-0.002	0.016	-0.001	*x*_22_	0.000	-0.045	-0.003
*x*_6_	0.001	-0.016	0.000	*x*_23_	0.000	-0.036	-0.002
*x*_7_	-0.001	0.002	0.001	*x*_24_	0.000	0.032	0.002
*x*_8_	-0.002	0.005	0.000	*x*_25_	-0.001	0.034	-0.002
*x*_9_	-0.004	-0.072	-0.002	*x*_26_	0.005	0.016	-0.002
*x*_10_	0.000	0.019	0.002	*x*_27_	-0.002	-0.088	-0.002
*x*_11_	-0.001	-0.004	0.000	*x*_28_	0.001	-0.017	0.001
*x*_12_	-0.002	0.010	0.000	*x*_29_	-0.002	-0.047	-0.003
*x*_13_	0.005	0.027	-0.002	*x*_30_	-0.002	0.019	0.001
*x*_14_	-0.001	0.013	0.001	*x*_31_	0.000	-0.056	-0.003
*x*_15_	0.000	-0.054	-0.003	*x*_32_	0.001	-0.016	-0.003
*x*_16_	0.005	-0.015	0.000	*x*_33_	0.006	0.007	-0.002
*x*_17_	-0.003	-0.067	-0.002	*x*_34_	-0.003	0.006	0.000

This result indicates that the C3I is partially shaped by its own previous values. We speculate that people may extrapolate their present confidence to an assessment of future economic confidence, in addition to other relevant information.

The first part of Eq (4), i.e. *t* ≤ 47 corresponds to the period before December 2009. Matrix A, shown in Table 11, can be split into 2 categories of topics, namely those that contribute positively to C3I and those that contribute negatively according to their coefficients. Note that the topics themselves do not contribute to C3I. The attention they receive in the population, measured by Google trends volume, is used as an indicator of the population’s pre-occupation with the topic in relation to the C3I. The topics in Table 11 thus reveal the internal topical structure of this particular measurement of consumer confidence through a behavioral measure and which topics contribute negatively or positively to our estimation of C3I. As shown in Tables 11, 12, and 13 we see that a number of topics that contribute positively to our estimation of C3I change polarity after November 2009. This change may indicate that the population changed its assessment of these topics, leading to a different contribution to their consumer confidence, or potentially a change in how the CCI is measured. For example, when a large number of individuals search for “over capacity” this might occur because of the perception of over capacity as a negative issue, while some years later, people might search for the same topic from the position that over capacity is improving, hence making a positive contribution to their consumer confidence.

**Table 11:** Matrix *A*: Parameters of topics’ current effect on C3I before December 2009.

Positive Topics	Influence to C3I	Negative Topics	Influence to C3I
CPI	0.006	Economic performance	-0.002
Deposit reserve rate	0.005	Demand	-0.002
Stocks	0.005	Foreign exchange	-0.002
Inflation	0.005	Private investment	-0.002
Trade balance	0.005	Employment	-0.002
GDP growth rate	0.001	US. economy	-0.003
Income gap	0.001	Consumption	-0.003
Urbanization	0.001	Economy transition	-0.003
PPI	0.000	Real economy	-0.004
Investment	0.000	Over capacity	-0.005

We list the ten most positive and ten most negative topics with respect to their effect on C3I in our model (the same as Matrix *B* and *C*).

**Table 12:** Matrix *B*: Parameters of topics’ current effect on C3I after November 2009.

Positive Topics	Influence to C3I	Negative Topics	Influence to C3I
Over capacity	0.081	Urbanization	-0.017
Housing price	0.034	Food price	-0.036
Real estate sales	0.032	Investment	-0.036
Inflation	0.027	Exchange rate	-0.045
Tax	0.019	Crude oil price	-0.047
Fixed investment	0.019	Interest rate for loan	-0.054
Population aging	0.019	PPI	-0.056
Private investment	0.016	US. economy	-0.067
Deposit reserve rate	0.016	Real economy	-0.072
International trade(import and export)	0.013	Foreign exchange	-0.088

**Table 13:** Matrix *C*: Parameters of topics’ future effect on C3I.

Positive Topics	Influence to C3I	Negative Topics	Influence to C3I
Real estate sales	0.002	Foreign exchange	-0.002
Population aging	0.002	Real economy	-0.002
Tax	0.001	GDP growth rate	-0.003
International trade(import and export)	0.001	PPI	-0.003
Urbanization	0.001	Economy transition	-0.003
Economic performance	0.001	Exchange rate	-0.003
Employment situation	0.001	Dollar trend	-0.003
Fixed investment	0.001	Interest rate for loan	-0.003
Demand	0.000	Crude oil price	-0.003
Trade balance	0.000	Food price	-0.003

Matrices *A*, *B*, and *C* indeed reveal significant changes in the structure of the C3I over time. In Tables 11, 12 and 13, we show how certain topics contribute positively or negatively to C3I values. In particular we see that before December 2009 (Table 11) positive topics include “stocks”, “CPI”, and “trade balance”. Negative topics notably include “prices”, e.g. “housing”, “fuel”, “food”, “over capacity”, and concerns about “economic transition”. Examining Table 12 we find that these are not influencing C3I as strongly after November 2009. Rather, the top ranked positively contributing topics are now “over capacity”, “real estate”, and “housing prices”. We do note that the negatively contributing topics continue to include “exchange rates” and “foreign exchange”.

Comparing Tables 11 and 12 with 13 reveals that the “future” influence of topics in our C3I model might overall be less than its current influence. Positive topics such as “real estate sales”, “population aging” and negative topics such as “crude or food price”, “exchange rate” have much lower parameter values in Table 13. In addition, these results shows that social media influence on C3I in our model increases after November 2009 possibly indicating that the public is increasingly expressing their outlook through online activity.

**Fig 6:**
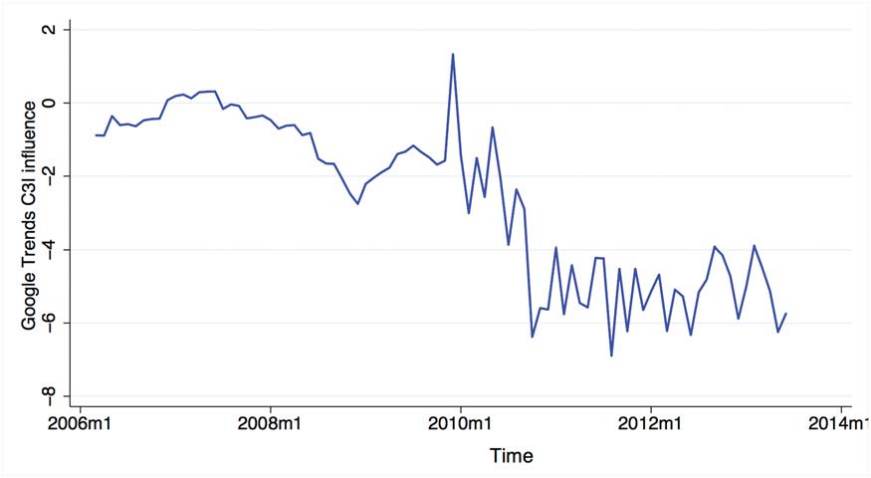
The contribution of Google Trends Data to our C3I model plotted over time reveals a downward trend possibly indicating that the public are losing economic confidence as judged from search engine queries.

As shown in Fig 6, our Google Trends data indicates a consistent downward trend in consumer confidence from 2007 to the present which is not mirrored by official CCI data. However, Google Trends data presumably provides only a partial indicator of the factors that shape consumer confidence. We can therefore not conclude that our Google Trends model indicates an actual downtrend in consumer confidence. This result does point to an interesting divergence between two different, but related measures of consumer confidence. We also note that after the observed discontinuity, CCI does exhibit a slight downward trend.

**Fig 7:**
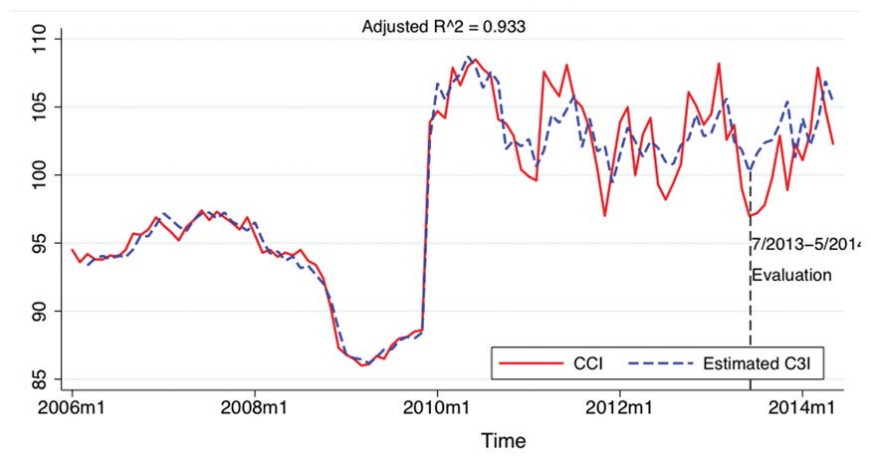
Graph overlay of CCI values estimated by our C3I model vs. official CCI values reported by the National Bureau of Statistics of China. Note that the period from July 2013 to May 2014 represents an estimation of CCI values on the basis of *new* Google trends data not included in the data used to generated our model to determine model robustness.

Finally, we compare C3I values generated by our model to the actual CCI values in Fig 7 which highlights the strong degree of correspondence between our model and actual CCI values as reported by the Chinese National Bureau of Statistics. In fact, after conducting our original analysis, we obtained new Google Trends data for the period July 2013 to May 2014, nearly a year, and re-applied the model developed from the original to this new Google Trends data. As shown in Fig 7 our model outcomes match the new C3I values quite well, in spite of the renormalization that Google applies to each new data request, indicating that the C3I model is robust to minor changes in the underlying Google Trends data.

## Conclusions

We model Chinese Consumer Confidence by analyzing the relationship between Chinese CCI data and Google Trends time series for query topics derived from official CCI questionnaires. Our model manages to approximate historical CCI values as well as new C3I values obtained after analysis of the original data by relying merely on Google Trends data as well as lagged CCI data manages. This finding suggests that the results of expensive and time-consuming Consumer Confidence surveys might be complemented by more economical and time-efficient methods that leverage online behavioral indicators and may additionally reveal the deeper structure of the abstract notion of consumer confidence as measured by the CCI. In fact, rather than an approximation of official CCI data, the use of Google Trends data might in fact complement the assessment of consumer confidence by incorporating additional relevant dimensions of consumer confidence and avoiding structural measurement changes.

We do caution that our use of the CCI as ground truth implies that any biases or deficiencies of the CCI will impact the validity of our own model as well. By extracting separate terms and topics from the CCI’s survey questions we may not fully capture the essence of how it semantically expresses Chinese consumer confidence. Furthermore, our selection is restricted to the CCI and may thus not comprehensively capture the full extent of actual Chinese consumer confidence. Future work will be directed at a more exhaustive and principled translation of the notion of consumer confidence to a set of search engine query terms, possibly from a variety of other sources. In fact, our reliance on Google trends data may introduce a number of other issues. If a particular aspect of consumer confidence can not be gauged from search engine volume, our method won’t capture it. As suggested by [34] the validity and accuracy of our model could thus be improved by the inclusion of other related indicators of consumer behavior such as social media feeds, blog volume, newspaper data, etc.

In spite of the deficiencies of our present approach, we have demonstrated the feasibility of modeling large-scale socio-economic phenomena such as consumer confidence from behavioral online data such as Google search queries. This opens new possibilities for more exhaustive, accurate, and finer-grained models of complex dynamic socio-technical systems such as a nation’s economy which is shaped by the interactions of large number of autonomous agents that respond to individual and collective conditions, as well as global systemic information such as financial news, economic growth forecasts, GDP numbers, and inflation numbers.

## Supporting Information

S1 Table Translation of ECQ Questionnaire with model topics marked in bold. (PDF)
