# Supplementary material for: Computational Models of Consumer Confidence from Large-Scale Online Attention Data: Crowd-Sourcing Econometrics
Source: PLoS One. 2015 Mar 31;10(3):e0120039. doi: 10.1371/journal.pone.0120039 (PMC4380490; doi:10.1371/journal.pone.0120039)
Supplement: S1 Table — (PDF) [file pone.0120039.s001.pdf]

Translation of ECQ Questionnaire with model topics marked in bold.

Question	Variables	Question & Topics
Q1	x_{40}, x_{12}, x_{34}, x_{23}	What is your judgment on the following aspects of China's economic operation? A) **Macro-economy** B) **Demand** C) **Investment** D) **Consumption**
Q2	x_4	How do you think Chinese current **economic performance**?
Q3	x_{14}	What do you think the next six months of **imports and exports** growth will become?
Q4	x_3	What do you feel the next six months, **China's foreign trade balance** will be?
Q5	x_{32}	You expect 2013 annual **GDP growth rate** will be:
Q6	x_{33}	What do you consider the next six months of **CPI** will be?
Q7	x_{31}	What do you think the next six months of **PPI** will be?
Q8	x_{29}, x_{20}	What do you think of the **international crude oil** and **food prices** over the next six months will be?
Q9	x_9	What the current liquidity situation of the **real economy** is in your eyes?
Q10	x_{26}	What do you think the next six months **deposit reserve rate** should be?
Q11	x_{15}	What do you feel the next six months **interest rate for loan** should become?
Q12		What do you think the current for the following currencies into RMB nominal exchange rate is in: A) Dollar B) Euro C) Pound D) Yen
Q13	x_{18}	How do you think the **dollar value may change** in the next 6 months?
Q14	x_{35}	What do you think the **RMB against the U.S. dollar** will become in the next six months?
Q15	x_{27}	What do you think the **foreign exchange** balance of China will be in the next six months?
Q16	x_{16}	What do you think the domestic **stock** market prices in the next three months will be?
Q17	x_{41}	What do you think the growth of direct **foreign investment** will be?
Q18	x_{30}	How do you think China's **fixed asset investment** growth will be? What would be the growth rate of whole year?
Q19	x_{38}	You believe the investment in **real estate development** in 2013 will increase ? over the same period of the previous year.
Q20	x_{25}	How do you think the trend of **housing price** for the next six months will be?
Q21	x_{24}	How do you think the **real estate sales** over the next six months will be?
Q22	x_{17}	What?s your idea concerning ? next six months the trend of the **U.S. economy**?
Q23		What do you think the European debt crisis situation will be in the next six months?
Q24	x_{36}	How do you think the **exchange rate of Japanese Yen against US dollar** in the next six months will be?
Q25	x_7, x_8	How do you think the **employment situation** of China this year ?
Q26	x_{44}	In response to the declining trend to the **labor force**, what measures can be taken in your opinion?
		(1) to promote agricultural moderate scale of operations, improve the efficiency of land productivity
	x_{23}	(2) to increase **investment** in education , improve population quality
		(3) to encourage various technological innovation,and improve total factor productivity
		(4) to intensify reform efforts to further reform the bonus release

Continued on previous page...

Table S1 *Continued from previous page*

Question	Variables	Question & Topics
	x_{10}	(5) to make the **family planning policy** to respond appropriately adjust to demographic changes
		(6) to moderate lower economic growth
	x_{19}	(7) to accelerate the pace of **economic transition**
		(8) Others (please specify) :
Q27		Comparing with the last 6 months, what do you think the local government funding in the next half year?
Q28	x_{39}	What's your judgment to local government **debt risk**?
Q29		Aiming at the local fiscal revenue growth becoming slow, the cumulative risk of local financing becoming heavy, what measures do you think should be taken?
		(1) to determine the reasonable revenue growth target
	x_{42}	(2) to make a **reduction of administrative expenses**
		(3) to bind government investment to related entities
	x_5	(4) to expand the participation of **private investment**
	x_{21}	(5) to improve the **tax** system , increase local property rights
		(6) to open up new sources of revenue (such as resource tax, housing property tax , etc.)
	x_1	(7) to give the **local government permission of issued debt**
		(8) to improve income distribution mechanism of state-owned monopoly
	x_{37}	(9) to appropriate to relax regulation of **real estate**
		(10) Others (please specify) :
Q30		What is the biggest risk facing the Chinese economy in 2013 in your opinion?
	x_{13}	(1) **Inflation**
		(2) decline in economic growth
	x_{19}	(3) the slow progress in **economy transition**
	x_{43}	(4) the blind **expansion of investment**
	x_{28}	(5) ignore the quality in the **urbanization process**
	x_{25}	(6) significant fluctuations in **housing prices**
	x_{44}	(7) **labor** shortages
		(8) the risk of local financing
		(9) private lending risk
	x_2	(10) Exacerbated by **overcapacity**
		(11) Larger pressure on energy saving and emission reduction
		(12) the degradation of export
		(13) ineffective environmental resources protection
	x_6	(14) further deterioration to the **income gap**
	x_{11}	(15) operational difficulties for **small and medium-sized enterprise**
		(16) Others(please specify) :
Q31		31. What is your suggestion to the further macroeconomic policies and reforms?
